# Editorial: Chromatin Regulation in Cell Fate Decisions

**DOI:** 10.3389/fcell.2021.734020

**Published:** 2021-09-01

**Authors:** Justin Brumbaugh, Bruno Di Stefano, José Luis Sardina

**Affiliations:** ^1^Department of Molecular, Cellular, and Developmental Biology, University of Colorado Boulder, Boulder, CO, United States; ^2^Molecular, Cellular and Developmental Biology, University of Colorado Comprehensive Cancer Center, Aurora, CO, United States; ^3^Charles C. Gates Center for Regenerative Medicine, University of Colorado Anschutz Medical Campus, Aurora, CO, United States; ^4^Department of Molecular and Cellular Biology, Baylor College of Medicine, Houston, TX, United States; ^5^Center for Cell and Gene Therapy, Baylor College of Medicine, Houston, TX, United States; ^6^Stem Cells and Regenerative Medicine Center, Baylor College of Medicine, Houston, TX, United States; ^7^Epigenetic Control of Haematopoiesis Group, Josep Carreras Leukaemia Research Institute, Barcelona, Spain

**Keywords:** chromatin, gene regulation, epigenetics, pluripotency, differentiation, cell fate, development, stem cells

Cell fate decisions, including events that occur naturally (e.g., development/embryogenesis, differentiation, regeneration, homeostasis) and experimentally (e.g., reprogramming to induced pluripotent stem (iPS) cells, directed differentiation, transdifferentiation), are typically mediated by transcription factors in concert with epigenetic modifications (Apostolou and Hochedlinger, 2013). These crucial regulators direct gene expression changes to establish cell-type specific transcriptional profiles. Mechanistically, a wide range of chromatin-related processes are involved (DNA and histone modifications, histone variants, genome topology, RNA processing and many more). The interplay between all of these layers of epigenetic regulation finally controls the transcriptional output of the conversion process, thus determining the final cell fate. This special edition of Frontiers in Cell and Developmental Biology examines the role of chromatin-based regulation in controlling cell fate decisions.

Many epigenetic marks dramatically change during embryogenesis, suggesting that they have fundamental roles in developmental decisions. For example, the early embryo experiences an initial, widespread loss of DNA methylation that is reacquired around the time of implantation. In this edition, Greenberg reviews the mechanisms responsible for these dynamics, with a focus on key regions that escape DNA methylation during its reestablishment. It remains unresolved whether this early round of DNA demethylation is passive (i.e., DNA methylation is diluted over multiple rounds of cell division) or active (i.e., resulting from enzymatic action). In any case, the Ten-eleven translocation (TET) class of DNA demethylases, and specifically TET2, are crucial for exiting pluripotency and initiating differentiation (Dai et al., 2016). Garcia-Outeiral et al. provide an updated perspective on TET2's role in these processes as well as reprogramming, and discuss the possibility that TET2 is also responsible for oxidizing RNA methylation. Together, these publications highlight the importance of DNA/RNA (de)methylation in the early embryo.

Pluripotent stem cells (PSCs) provide an experimentally tractable system to study the association between chromatin and cell fate decisions. In recent years, multiple cell culture conditions were established to maintain self-renewing PSCs (both mouse and human) but with distinct molecular and phenotypic properties. Although all of the culture conditions support pluripotency, each is believed to represent a different developmental stage and collectively, these tools have broadened our ability to model early embryonic development. Correspondingly, cells cultured in each condition have their own epigenetic signature and transitioning between states leads to large rearrangements in the chromatin landscape. Sun et al. summarize the recent characterization of these states and also compare culture conditions and epigenetic regulation in mouse and human. Work in PSCs has also shed light on the proteins responsible for modifying chromatin during developmental transitions. Franklin et al. provide a comprehensive review of histone chaperones and their role in cell fate specification, with a focus on proteins responsible for depositing histone H3. Once deposited, histones are further regulated by nucleosome remodelers, many of which have been linked to development and differentiation. Separate articles by the Ye et al. and Pagliaroli and Trizzino labs lay out the role of mammalian SWI/SNF complexes in pluripotency, differentiation and developmental disorders. Cis-regulatory elements are likewise important in guiding cell fate decisions. Agrawal and Rao discuss the contribution of super-enhancers and CTCF-mediated three-dimensional genome organization in regulating cell-type specific expression programs. These articles provide important insight into the role of chromatin in directing embryonic cell fate decisions.

During the formation and maintenance of adult tissues, several molecular mechanisms coordinate gene expression programs to ensure proper lineage specification. For example, chromatin organization is tightly controlled by different epigenetic regulators during brain development. In this issue, Mastrototaro et al. show that the nuclear receptor TBL1XR1 modulates the stability of the epigenetic repressive complex NCOR, thus impacting neural progenitor self-renewal and differentiation. DNA methylation at CpG residues is another important regulator of neuronal development. Recently, non-CpG DNA methylation (mCH) was detected in the vertebrate nervous system (de Mendoza et al., 2021); yet, its biological relevance remains unclear. Ross et al. found that mCH accumulates at gene bodies and transposable elements during zebrafish brain development and potentially plays a role in regulating the transcription at these genomic regions. Similar to its role in controlling pluripotency, TET-mediated DNA demethylation is a prominent mechanism of chromatin-based regulation in brain development and function. MacArthur and Dawlaty discuss the role of TET enzymes in neural differentiation and review how TET dysregulation contributes to neurological disorders.

As in the brain, differentiation of hematopoietic stem cells to specialized cells is accompanied by extensive chromatin remodeling. Interestingly, genes coding for regulators of DNA methylation (i.e., *DNMT3A and TET2*) are frequently mutated in a wide range of hematological malignancies, suggesting that DNA methylation is key for establishment and maintenance of hematopoietic cell identity. In this edition, Tsiouplis et al. provide a comprehensive review on TET enzymes as key regulators of the immune system in homeostasis and in pathological conditions. Chromatin modifying complexes including Polycomb group (PcG) of proteins have also been involved in hematopoietic cell differentiation. In their primary research article, García-Montolio et al. show that the epigenetic regulator PHF19 (a PRC2-associated factor) controls the balance between proliferation and differentiation of erythroid progenitors. These articles expand on a growing research interest in correlation between hematopoiesis and chromatin regulation.

Unlike the brain or blood, current evidence suggests that the liver does not have a dedicated stem cell compartment to mediate homeostasis and regeneration. However, following injury or resection, hepatic cells undergo substantial changes in proliferative capacity and are capable of transdifferentiation or de-differentiation. Mediating these changes are a variety of epigenetic regulators. In this issue, Aloia discusses the role of epigenetic mechanisms in liver regeneration and expands on how alterations in these mechanisms lead to complex disorders, including cancer.

Finally, DNA and histone methylation is a key epigenetic mechanism that potently influences transcriptional outputs. DNA, RNA, and histone methyltransferases require S-Adenosyl methionine (SAM) as a donor for the methyl group. Therefore, SAM plays an essential role in regulating the chromatin status of cells, consequently linking methionine metabolism with cell fate. SAM generation is catalyzed by the Adenosylhomocysteinase (AHCY) that reportedly binds to chromatin in pluripotent stem cells (Aranda et al., 2019). Vizán et al. review the evolution and biochemical properties of AHCY across all living organisms and highlight its functions in homeostasis and disease.

Together, the manuscripts in this Research Topic provide an overview of the latest research on the impact of chromatin regulation in instructing a full spectrum of cell fate decisions, ranging from early embryogenesis to regeneration.

## Author Contributions

JB, BD, and JS contributed to the editing of this Research Topic. All authors contributed to the article and approved the submitted version.

## Conflict of Interest

The authors declare that the research was conducted in the absence of any commercial or financial relationships that could be construed as a potential conflict of interest.

## Publisher's Note

All claims expressed in this article are solely those of the authors and do not necessarily represent those of their affiliated organizations, or those of the publisher, the editors and the reviewers. Any product that may be evaluated in this article, or claim that may be made by its manufacturer, is not guaranteed or endorsed by the publisher.
